# Cataract in patients with diabetes mellitus—incidence rates in the UK and risk factors

**DOI:** 10.1038/s41433-017-0003-1

**Published:** 2018-02-01

**Authors:** Claudia Becker, Cornelia Schneider, Samuel Aballéa, Clare Bailey, Rupert Bourne, Susan Jick, Christoph Meier

**Affiliations:** 1 Basel Pharmacoepidemiology Unit, Division of Clinical Pharmacy and Epidemiology, Department of Pharmaceutical Sciences, University of Basel, Basel, Switzerland; 2 Hospital Pharmacy, University Hospital Basel, Basel, Switzerland; 3 Creative Ceutical Ltd, London, UK; 4 University Hospitals Bristol NHS Foundation Trust, Bristol, UK; 5 Vision and Eye Research Unit, Postgraduate Medical Institute, Faculty of Medical Science, Anglia Ruskin University, Cambridge, UK; 6 Boston Collaborative Drug Surveillance Program, Boston University School of Public Health, Lexington, MA USA; 7 Boston Collaborative Drug Surveillance Program, Lexington, MA USA

**Keywords:** Risk factors, Diabetes complications, Lens diseases, Epidemiology

## Abstract

****Aims**:**

To analyze the risk of incident cataract (diagnosis or extraction) in patients with or without diabetes focusing on other comorbid conditions, antidiabetic drug use, and diabetes duration.

****Methods**:**

The study population comprised newly diagnosed diabetes patients (≥40 years) from the UK-based Clinical Practice Research Datalink (CPRD) between 2000 and 2015, and a random sample of the general population matched for age, sex, general practice, and year of diabetes diagnosis. We assessed cataract incidence rates (IRs) and performed a nested case-control analysis in the diabetic cohort to assess potential risk factors for a cataract.

****Results**:**

There were 56,510 diabetes patients included in the study. IRs of cataract were 20.4 (95% CI 19.8–20.9) per 1000 person-years (py) in patients with diabetes and 10.8 (95% CI 10.5–11.2) per 1000 py in the general population. IRs increased considerably around the age of 80 years and with a concomitant diagnosis of macular edema. The incidence rate ratio (IRR) was highest in patients of the age group of 45–54 years. In the nested case-control study, we identified 5800 patients with cataract. Risk of cataract increased with increasing diabetes duration (adj. OR 5.14, 95% CI 4.19–6.30 diabetes for ≥10 years vs. diabetes <2 years).

****Conclusions**:**

According to our study, diabetes is associated with an approximately two-fold increased detection rate of cataract. The risk of cataract associated with diabetes is highest at younger ages. Patients with diabetic macular edema are at an increased risk for cataract as well as patients with long-standing diabetes.

## Introduction

Cataract is the primary cause of blindness worldwide [1]. It is defined as a decrease in the transparency of the crystalline lens and can be further differentiated into nuclear, cortical, or posterior subcapsular cataract (PSC) [2]. Main risk factors in the developed world, besides advanced age, appear to be smoking [3], exposure to sunlight [4], and use of corticosteroids [5, 6]. A potential association between female gender and cataract remains controversial [7, 8].

Several studies have reported diabetes as a risk factor for cataract [9]. However, there are only few studies conducted with data from the UK [10–13], and only one previous study from the 1980s reported on incidence rates of cataract in a diabetic population [11].

The aim of this study was to assess incidence rates of cataract (diagnosis or extraction, subsequently named “diagnosis” throughout the manuscript) in patients newly diagnosed with diabetes and treated medically (aged 40 years or older at the time of the first diabetes diagnosis), and to compare them with individuals without diabetes from the general population. Furthermore, we aimed to quantify comorbid conditions, as well as prior diabetes medication use in diabetic patients with cataract, and we explored the association between diabetes duration, diabetic control, and cataract diagnosis risk.

## Methods

### Data source

We performed a retrospective observational study using data from the Clinical Practice Research Datalink (CPRD). This database provides health care information on some 10 million patients in the UK and has been previously described in detail [14, 15]. General practitioners (GPs) record information on demographics, diagnoses, and drug prescriptions, as well as on patient referrals and hospital admissions, using standardized coding systems, the READ codes. The Read clinical terminology system includes occupation; social circumstances, clinical signs and symptoms, laboratory tests and results, diagnoses, diagnostic, therapeutic or surgical procedures performed, as well as administrative items. The GPs generate prescriptions directly with the computer, and this information is automatically transcribed into individual computerized patient records. In addition, the CPRD records information on body mass index (BMI) and lifestyle variables including alcohol consumption and smoking. Recorded information on drug exposure and on diagnoses has been validated repeatedly and has proven to be of high quality [16, 17]. The CPRD currently covers about 7% of the UK population, and enrolled patients are representative of the UK with regard to age, sex, and geographic distribution [18]. The CPRD is managed by the Medicines and Healthcare products Regulatory Agency (MHRA) in the UK. The study protocol was reviewed and approved by the Independent Scientific Advisory Committee for MHRA database research (ISAC, reference number 16_065) and was made available to the journal reviewers. The investigators had access to anonymous information only.

### Study design

We first calculated incidence rates (IRs) of cataract (defined as (a) a cataract diagnosis or a recorded cataract surgery and (b) cataract surgery only) in patients with a first-time diagnosis of diabetes mellitus, compared to matched diabetes-free controls. We also assessed IRs in subgroups of diabetes patients with a diagnosis of macular edema or retinopathy at any time in their patient records.

In addition, we conducted a nested case-control analysis which we restricted to patients with diabetes, to further study diabetes-associated potential risk factors for a cataract diagnosis.

### Study population

To be considered a diabetic patient, an individual must have had a READ code for diabetes mellitus plus two or more prescriptions for medications for diabetes recorded within 6 months prior to and until 1 year after the first-time diagnosis of diabetes. The date of the first recording of either the diabetes diagnosis or the prescription for a diabetes medication was considered the start of follow-up. Patients had to be 40 years or older at the time of the diabetes diagnosis to be included. We matched diabetes-free patients from the comparison group to diabetic patients on age, sex, GP, and year of diabetes diagnosis (i.e., we followed diabetes-free patients from the same year on as the matched patient with diabetes). The study period was January 2000 to December 2015.

All individuals included in the study population were required to have a minimum of 3 years of medical history in the database prior to either the first diabetes diagnosis, or—in the comparison group—before the start of follow-up to contribute person-time.

We excluded individuals with a diagnosis (at any time in the record) of congenital cataract, cancer (except non-melanoma skin cancer), HIV, or alcoholism, patients with a diagnosis of glaucoma, glaucoma surgery, one or more prescriptions for drugs to treat glaucoma, traumatic or secondary cataract, as well as individuals with binocular blindness, cataract, or cataract extraction recorded before the start of follow-up.

### Outcome definition

We defined cataract patients as those with a READ code for cataract (diagnosis or cataract extraction) entered in the medical records by the GP.

### Covariates

In the nested case-control analysis, we assessed previous drug prescriptions for antidiabetic drugs prior to the cataract, categorized by the number of prescriptions before the index date (i.e., the cataract diagnosis or extraction date). Additional covariates of interest were diabetes duration and diabetic control, expressed as the average HbA1c level of the recordings during the last 3 years preceding the index date. We also assessed the distribution of HbA1c levels in diabetic cataract cases with or without the use of insulin. Furthermore, we assessed smoking status (never, ex-smoker, current, or unknown), body mass index (BMI < 25, 25–29.9, ≥30 kg/m^2^, unknown), and by means of READ codes various recorded comorbidities such as cardiovascular diagnoses (ischemic heart disease, congestive heart failure, stroke, or hypertension), as well as asthma/COPD or hyperlipidemia. We included all recordings prior to the cataract diagnosis/surgery in the definition of the comorbid status.

### Statistical analysis

We calculated incidence rates of first-time cataract separately for the diabetic cohort and for the matched individuals from the diabetes-free population, stratified by age (40–44, 45–49, 50–54, …, and ≥90 years), sex and calendar year of cataract occurrence. We computed the person-years at risk individually for each person in the study population. We assessed the person-time from the date of entry into the study until the patient had a cataract or any of the exclusion criteria recorded, the patient left the CPRD, died, or the study ended in December 2015, whichever came first. We used incident cases of cataract as the numerator, and the sum of person-years in the study population as the denominator within age, sex, and calendar year strata. For the subgroup analyses, we calculated person-time in patients with diabetic retinopathy or macular edema (defined by READ diagnoses codes), and we assessed the number of incident cataract cases in those groups, using the same stratification as for the total sample.

For the nested case-control analysis we only included patients with diabetes. In addition to diabetic cases with an incident cataract during follow-up, we identified from among the diabetic portion of the study population a control group of patients without cataract. The date of the first-time recording of a cataract was defined as the “index date”. We matched controls to cases by risk set sampling on year of birth, sex, index date, and years of history on the database. We attempted to find four control patients per case. We compared cases and controls with respect to the prevalence of diagnosed comorbidities, antidiabetic drug use prior to the cataract, as well as other covariates (see above) by conditional logistic regression analyses. We assessed the association between duration of diabetes history and the risk of cataract, and we included other covariates (as potential confounding factors) in the model to see if they changed the relative risk estimate (odds ratio) by >10%.

We conducted the analyses with the statistical software SAS (SAS Institute, Inc., Cary, NC, USA, version 9.4). Power calculation was performed before the conduct of the study to ensure adequate power to detect an OR of 1.2 or more for the association of metformin exposure and subsequent cataract development at a significance level of 5%. Incidence rates and relative risk estimates (odds ratios (ORs)) are presented with 95% confidence intervals (CI). For confidentiality reasons (as required by the Medicines and Healthcare products Regulatory Agency, MHRA), cells containing <5 patients are marked with “x” and not display the actual number of cases.

## Results

### Follow-up analysis

We identified 56,510 patients with a first-time diagnosis of diabetes (with two or more antidiabetic drugs prescribed within the predefined time period), and the same number of patients in the comparison group without diabetes. Mean age at start of follow-up was 60.1 years (SD 11.4 years).

Incidence rates and incidence rate ratios are displayed in Tables 1a and 1b. The incidence rates (IRs) of cataract increased considerably around the age of 70 years until very advanced age, with the highest IRs in the age category 85–89 years. The incidence rate ratio (IRR, the ratio of IRs between diabetics and non-diabetics), however, was highest in patients of the age group of 45–54 years. Incidence rates per year of cataract diagnosis did not vary much during the study period (results not shown). The incidence rate of cataract diagnosis in diabetes patients with a diagnosis of macular edema recorded at any time in their patient records was considerably higher than in the general diabetic population (59.0, 95% CI 49.4–68.6). The incidence rate of cataract diagnosis in diabetes patients with retinopathy appeared to be only slightly higher than in the overall diabetic population (26.3, 95% CI 25.1–27.4).

**Table 1a:** Incidence rates of cataract (diagnoses and surgery cases) in patients newly diagnosed with diabetes mellitus and in diabetes-free individuals (matched to diabetes-free patients on age, sex, year of diabetes diagnosis) and incidence rate ratios (stratified by sex and age)

	Patients with diabetes				Diabetes-free comparison group					
	Person-years	Cases	IR per 1000 person-years	95% CI	Person-years	Cases	IR per 1000 person-years	95% CI	IRR	95% CI
All	284887.4	5802	20.4	19.8–20.9	284953.5	3087	10.8	10.5–11.2	1.9	1.8–2.0
Men	153029.5	2524	16.5	15.9–17.1	148846.7	1267	8.5	8.0–9.0	1.9	1.8–2.1
Women	131857.9	3278	24.9	24.0–25.7	136106.8	1820	13.4	12.8–14.0	1.9	1.8–2.0
Age 40–44	9224.9	22	2.4	1.4–3.4	8675.2	6	0.7	0.1–1.2	3.4	1.4–8.5
Age 45–49	25840.3	70	2.7	2.1–3.3	24085.6	10	0.4	0.2–0.7	6.5	3.3–12.5
Age 50–54	37674.4	193	5.1	4.4–5.8	35173.7	31	0.9	0.6–1.2	5.8	4.0–8.5
Age 55–59	44047.4	324	7.4	6.6–8.2	42089.5	118	2.8	2.3–3.3	2.6	2.1–3.2
Age 60–64	46713.6	526	11.3	10.3–12.2	45310.4	199	4.4	3.8–5.0	2.6	2.2–3.0
Age 65–69	43022.1	860	20.0	18.7–21.3	43263.2	369	8.5	7.7–9.4	2.3	2.1–2.6
Age 70–74	34468.6	1111	32.2	30.3–34.1	36143.3	611	16.9	15.6–18.2	1.9	1.7–2.1
Age 75–79	24776.3	1295	52.3	49.4–55.1	27036.8	782	28.9	26.9–31.0	1.8	1.7–2.0
Age 80–84	12867.7	911	70.8	66.2–75.4	15319.3	601	39.2	36.1–42.4	1.8	1.6–2.0
Age 85–89	4836.9	403	83.3	75.2–91.5	5989.4	290	48.4	42.8–54.0	1.7	1.5–2.0
Age ≥90	1415.1	87	61.5	48.6–74.4	1867.0	70	37.5	28.7–46.3	1.6	1.2–2.2

*CI* Confidence interval, *IR* Incidence rate, *IRR* Incidence rate ratio

**Table 1b:** Incidence rates of cataract (surgery only) in patients newly diagnosed with diabetes mellitus and in diabetes-free individuals (matched to diabetes-free patients on age, sex, year of diabetes diagnosis) and incidence rate ratios (stratified by sex and age)

	Patients with diabetes				Diabetes-free comparison group					
	Person-years	Cases	IR per 1000 person-years	95% CI	Person-years	Cases	IR per 1000 person-years	95% CI	IRR	95% CI
All	380090.5	4699	12.4	12.0–12.7	289259.9	2289	7.9	7.6–8.2	1.6	1.5–1.6
Men	203269.4	1996	9.8	9.4–10.3	150734.5	913	6.1	5.7–6.4	1.6	1.5–1.8
Women	176820.9	2703	15.3	14.7–15.9	138525.4	1376	9.9	9.4–10.5	1.5	1.4–1.6
Age 40–44	11343.3	12	1.1	0.5–1.7	8698.1	2	0.2	0.0–0.5	4.6	1.0–20.6
Age 45–49	32796.2	44	1.3	0.9–1.7	24153.4	7	0.3	0.1–0.5	4.6	2.1–10.3
Age 50–54	48588.2	150	3.1	2.6–3.6	35271.8	19	0.5	0.3–0.8	5.7	3.5–9.2
Age 55–59	57183.9	246	4.3	3.8–4.8	42175.4	89	2.1	1.7–2.5	2.0	1.6–2.6
Age 60–64	61635.6	402	6.5	5.9–7.2	45651.3	132	2.9	2.4–3.4	2.3	1.9–2.8
Age 65–69	58201.5	624	10.7	9.9–11.6	43727.6	271	6.2	5.5–6.9	1.7	1.5–2.0
Age 70–74	47662.3	930	19.5	18.3–20.8	37014.7	438	11.8	10.7–12.9	1.6	1.5–1.8
Age 75–79	34797.3	1 085	31.2	29.3–33.0	27983.3	579	20.7	19.0–22.4	1.5	1.4–1.7
Age 80–84	18739.6	791	42.2	39.3–45.2	16114.6	468	29.0	26.4–31.7	1.5	1.3–1.6
Age 85–89	7172.8	333	46.4	41.4–51.4	6448.0	237	36.8	32.1–41.4	1.3	1.1–1.5
Age ≥90	1969.7	82	41.6	32.6–50.6	2021.8	47	23.2	16.6–29.9	1.8	1.3–2.6

*CI* Confidence interval, *IR* Incidence rate, *IRR* Incidence rate ratio

### Nested case-control analysis among patients with diabetes

We included a total of 5800 cataract cases and 21,432 matched controls in the nested case-control analysis. Mean age of the cases and controls was 72.1 (±9.3) years. In total 50% of the cataract cases were women. The mean duration of medical history recorded in the CPRD before the index date was 15.8 (±5.1) years in cases and 16.0 (±5.0) years in controls.

The main characteristics of diabetic cataract cases and controls are displayed in Table 2. Current smokers compared with non-smokers, and obese patients compared with normal weight patients, were not at increased risk of developing cataract.

**Table 2:** Characteristics of cataract cases and controls, nested case-control analysis

		Cases (%) (*n* = 5800)	Controls (%) (*n* = 21432)	Crude OR* (95% CI)
Age (years)	40–49	92 (1.6)	328 (1.5)	−
	50–59	517 (8.9)	2000 (9.3)	–
	60–69	1386 (23.9)	5368 (25.1)	–
	70–79	2450 (41.5)	9111 (42.5)	–
	≥80	1399 (24.1)	4625 (21.6)	–
Sex	Male	2524 (43.5)	9333 (43.6)	–
	Female	3276 (56.5)	12,099 (56.5)	–
Smoking	Non-smoker	2227 (38.4)	8542 (39.9)	1.00 (ref)
	Current	621 (10.7)	2578 (12.0)	0.95 (0.85–1.05)
	Past	2899 (50.0)	10,080 (47.0)	1.12 (1.05–1.19)
	Unknown	53 (0.9)	232 (1.1)	0.78 (0.57–1.07)
BMI	<25	1046 (18.0)	3617 (16.9)	1.00 (ref)
	25–29.9	2083 (35.9)	7701 (35.9)	0.95 (0.87–1.04)
	≥30	2547 (43.9)	9563 (44.6)	0.96 (0.88–1.04)
	Unknown	124 (2.1)	551 (2.6)	0.69 (0.56–0.86)
CHF	No	5279 (93.5)	20,047 (93.5)	1.00 (ref)
	Yes	521 (9.0)	1385 (6.5)	1.39 (1.25–1.55)
IHD	No	4439 (76.5)	17,179 (80.2)	1.00 (ref)
	Yes	1361 (23.5)	4253 (19.8)	1.26 (1.17–1.35)
Hypertension	No	2020 (34.8)	7908 (36.9)	1.00 (ref)
	Yes	3780 (65.2)	13,524 (63.1)	1.10 (1.03–1.17)
Stroke/TIA	No	5144 (88.7)	19,094 (89.1)	1.00 (ref)
	Yes	656 (11.3)	2338 (10.9)	1.02 (0.93–1.12)
MI	No	5253 (90.6)	19,683 (91.8)	1.00 (ref)
	Yes	547 (9.4)	1749 (8.2)	1.18 (1.06–1.30)
Dyslipidemia	No	4218 (72.7)	16,257 (75.9)	1.00 (ref)
	Yes	1582 (27.3)	5175 (24.2)	1.22 (1.15–1.31)
Asthma	No	4765 (82.2)	17,927 (83.7)	1.00 (ref)
	Yes	1035 (17.8)	3505 (16.4)	1.13 (1.04–1.22)
COPD	No	5234 (90.2)	19,910 (92.9)	1.00 (ref)
	Yes	566 (9.8)	1522 (7.1)	1.42 (1.28–1.57)
HbA1c level**				
	<48 mmol/mol	1967 (33.9)	7570 (35.3)	1.00 (ref)
	48–57.9 mmol/mol	1615 (27.8)	6024 (28.1)	1.04 (0.97–1.12)
	58–68.9 mmol/mol	1059 (18.3)	3681 (17.2)	1.12 (1.03–1.22)
	≥69 mmol/mol	952 (16.4)	3148 (14.7)	1.16 (1.06–1.27)
	unknown	207 (3.6)	1009 (4.7)	0.73 (0.62–0.86)

*BMI* Body Mass Index, *CHF* congestive heart failure, *CI* confidence interval, *COPD* chronic obstructive pulmonary disease, *HbA1c* glycated hemoglobin, *IHD* ischemic heart disease, *MI* myocardial infarction, *OR* Odds ratio, *ref* reference, *TIA* transient ischemic attack

* adjusted for age, sex, index date, *GP* practice, and years of follow-up by matching

** average level in the last 3 years prior to the cataract diagnosis

The risk of a cataract diagnosis rose with higher HbA1c level and was 20% increased for the highest HbA1c level (test for trend, *p* < 0.0001). HbA1c of 58 mmol/mol or more was observed in 56.8% of insulin users and in 30.5% of diabetics without insulin therapy. Cataract diagnosis risk was considerably increased in patients with long-term steroid exposure (Table 3; ≥30 prescriptions: adj. OR 1.87, 95% CI 1.62–2.16). Diabetes duration was significantly associated with the risk of a cataract diagnosis (test for trend: *p* < 0.001). Antidiabetic drug treatment did not yield any clear associations with cataract diagnosis risk.

**Table 3:** Drug exposure and duration of diabetes history of cataract cases and controls, nested case-control analysis

	Cases (%)	Controls (%)	Crude OR* (95% CI)	Adj. OR** (95% CI)
Metformin				
None	860 (14.8)	3303 (15.4)	1.00 (ref)	1.00 (ref)
1–9 Rx	1337 (23.1)	5874 (27.4)	0.90 (0.82–1.00)	1.08 (0.98–1.21)
10–29 Rx	1518 (26.2)	6251 (29.2)	1.05 (0.95–1.16)	1.23 (1.10–1.37)
≥30 Rx	2085 (36.0)	6004 (28.0)	1.64 (1.48–1.82)	1.34 (1.19–1.50)
Sulfonylureas				
None	2940 (50.7)	12,824 (59.8)	1.00 (ref)	1.00 (ref)
1–9 Rx	867 (15.0)	2908 (13.6)	1.31 (1.20–1.43)	1.24 (1.13–1.35)
10–29 Rx	832 (14.3)	3031 (14.1)	1.24 (1.14–1.36)	1.06 (0.97–1.17)
≥30 Rx	1161 (20.0)	2669 (12.5)	2.09 (1.92–2.28)	1.37 (1.24–1.50)
Insulin				
None	5153 (88.8)	20,001 (93.3)	1.00 (ref)	1.00 (ref)
1–9 Rx	229 (4.0)	643 (3.0)	1.40 (1.20–1.63)	1.27 (1.08–1.50)
10–29 Rx	192 (3.3)	430 (2.0)	1.81 (1.52–2.16)	1.46 (1.22–1.76)
≥30 Rx	226 (3.9)	358 (1.7)	2.72 (2.29–3.24)	1.85 (1.53–2.23)
TZD				
None	4954 (85.4)	19,278 (90.0)	1.00 (ref)	1.00 (ref)
1–9 Rx	249 (4.3)	728 (3.4)	1.41 (1.21–1.63)	1.02 (0.87–1.19)
10–29 Rx	289 (5.0)	784 (3.7)	1.55 (1.35–1.79)	1.07 (0.92–1.24)
≥30 Rx	308 (5.3)	642 (3.0)	2.07 (1.80–2.40)	1.27 (1.09–1.48)
Other antidiabetics				
None	5254 (90.6)	20,055 (93.6)	1.00 (ref)	1.00 (ref)
1–9 Rx	231 (4.0)	634 (3.0)	1.48 (1.27–1.73)	1.11 (0.94–1.31)
10–29 Rx	193 (3.3)	505 (2.4)	1.62 (1.36–1.92)	1.13 (0.94–1.36)
≥30 Rx	122 (2.1)	238 (1.1)	2.18 (1.74–2.74)	1.30 (1.01–1.65)
Systemic steroids				
None	4118 (71.0)	16011 (74.7)	1.00 (ref)	1.00 (ref)
1–9 Rx	1064 (18.3)	3883 (18.1)	1.08 (1.00–1.16)	1.08 (0.99–1.16)
10–29 Rx	294 (5.1)	847 (4.0)	1.35 (1.17–1.54)	1.41 (1.22–1.63)
≥30 Rx	324 (5.6)	689 (3.2)	1.85 (1.61–2.13)	1.87 (1.62–2.16)
Duration of diabetes				
<2 years	2024 (34.9)	9482 (44.2)	1.00 (ref)	1.00 (ref)
2–4.9 years	1805 (31.1)	7198 (33.6)	1.43 (1.32–1.54)	1.24 (1.13−1.36)
5–9.9 years	1551 (26.7)	4174 (19.5)	2.76 (2.52–3.04)	2.05 (1.81−2.31)
≥10 years	420 (7.2)	578 (2.7)	8.03 (6.74–9.57)	5.14 (4.19−6.30)

* adjusted for age, sex, index date, GP practice, and years of follow-up by matching

** additionally adjusted for the variables in the table

*TZD* Thiazolidinediones, *Rx* prescriptions, *OR* Odds ratio, *CI* confidence interval, *ref* reference

## Discussion

This large observational study provides evidence for an increased risk of cataract diagnosis in patients with diabetes compared to a diabetes-free control group. This trend stayed the same if cataract cases were defined as either having a mere cataract diagnosis or a cataract surgery (Table 1a) or if only cases with cataract surgery were considered (Table 1b). The incidence rates of cataract diagnosis in diabetic patients with a diagnosis of macular edema were considerably higher than in the general diabetic population. Incidence rates of cataract diagnosis in diabetics with retinopathy appeared to be only slightly higher than in the overall diabetic population.

There has only been one previous study reporting incidence rates of cataract in diabetic patients in the UK [11]. This study, however, used data collected at visits of outpatient clinics, and some data were derived from self-completed questionnaires by the patients between 1979 and 1992. The authors were in a position to distinguish between type 1 diabetes (“insulin dependent”) and type 2 diabetes, T2DM, (“non-insulin treated” and “insulin-treated non-insulin dependent diabetes”). Our study sample most likely encompassed only individuals with type 2 diabetes (since a first-time diabetes diagnosis was required to have been recorded after the age of 39 years), but we did not differentiate between insulin-treated and non-insulin treated type 2 diabetes. Nevertheless, both studies found a higher risk of cataract in diabetic women than men. The incidence rates of cataract diagnosis in our study seemed to be slightly higher (overall 20.4 per 1000 person-years (py) vs. 11.7 and 17.8 per 1000 py, for non-insulin treated T2DM and insulin-treated T2DM respectively, in the study by Janghorbani et al. [11]. Both studies observed increased rates of cataract in the subgroup of diabetic patients with retinopathy. Furthermore, both studies found a statistically significant positive trend for the association between diabetes duration and risk of cataract, and both yielded increased relative risks for cataract with higher HbA1c levels. An increased risk of cataract for diabetic women compared to men has also previously been reported in the UK (relative risk of 7.85, 95% CI 4.30–14.3, in women vs. RR of 3.42, 95% CI 2.05–5.70 in men) [10]. This study also reported a higher risk for diabetic cataract in the younger age groups (RR of 12.6, 95% CI 2.76–57.9, for age 50–59, RR of 5.56, 95% CI 2.74–11.3, for age 60–69, and RR of 4.20, 95% CI 2.58–6.83, for age 70–79), a finding which corresponds to the higher IRRs in individuals below the age of 55 years observed in the current study. Thus, our data are consistent with findings from previous studies reporting that cataract development occurs more frequently at an earlier age in diabetes patients compared to diabetes-free controls.

Outside the UK, the Wisconsin Epidemiologic Study of Diabetic Retinopathy investigated the incidence of cataract extraction in people with diabetes [19]. Age (OR of 1.79, 95% CI 1.47–2.18) and use of insulin (OR of 2.11, 95% CI 1.43–3.11) were associated with increased risk of cataract in patients with type 2 diabetes. We also observed an increased risk of cataract diagnosis in insulin users. The higher percentage of poor glycemic control in insulin users compared with diabetics without insulin therapy may be an explanation for this observation (59.4% vs. 31.7% of patients with cataract and HbA1c ≥ 58 mmol/mol).

Our study has several limitations. Because cataract is developing slowly over a period of time, the date of the initial recording of cataract (or cataract surgery), i.e., the index date, does not equal the actual cataract onset. Therefore, assessing the association of cataract with previous exposure to diabetes medication or with diabetes duration up to the index date remains somewhat arbitrary. Furthermore, we were not in the position to differentiate between the three types of cataract, as the diagnosis codes most often used by the GPs were general cataract codes. In addition, diabetic patients receive regular eye checks from the hospital eye service whereas in the general population, detection of early cataracts with no impact on vision may not necessarily be fed back to the GP by the optometrist if mild and not requiring intervention. Thus, there may be a slight ‘over-reporting’ in the diabetes group compared to the general population who are not having regular eye checks. Our incidence rates may therefore rather reflect detection rates as this was an observational study and no intervention study. In addition, we did not perform a case validation on cataract diagnosis cases for this study; however, a recent validation study by Kang et al. [20] of cataract codes in the CPRD found a positive predictive value for the cataract code algorithm of 92.0% (90.3–93.7%). To increase the likelihood of studying true diabetes patients, we only included diabetic patients who received medical treatment within a predefined time frame around the diabetes diagnosis.

Our study has several strengths. The data source is a well-established primary care database of high quality and completeness. The information on drug exposure and diagnoses was recorded prospectively and independent of a study hypothesis, thereby recall bias could not have influenced our results. Furthermore, by excluding all patients with <3 years of recorded history in the database prior to the first-time diagnosis of diabetes, we minimized the risk of including prevalent diabetes cases. We were able to incorporate many potential confounders in our analysis, such as BMI, smoking, a range of comorbid conditions and prescriptions for antidiabetic drugs.

In conclusion, this large observational study demonstrates that incidence rates of cataract diagnosis in patients with diabetes are higher than among diabetic-free patients, particularly at younger age. The overall approximately twofold increased risk of cataract diagnosis associated with diabetes increases with diabetes duration. Patients with diabetic macular edema are at an increased risk for a cataract diagnosis.

### Summary

#### What was known before

- Diabetes is a known risk factor for cataract.
- There are only few studies conducted on that topic with data from the UK, and only one previous study from the 1980s reported on incidence rates of cataract in a diabetic population.

#### What this study adds

- IRs of cataract were 20.4 (95% CI 19.8–20.9) per 1000 person-years (py) in patients with diabetes and 10.8 (95% CI 10.5–11.2) per 1000 py in the general population.
- IRs increased considerably around the age of 80 years and the incidence rate ratio (IRR), was highest in patients of the age group of 45–54 years.
- Cataract risk increased with increasing diabetes duration (adj. OR 5.14, 95% CI 4.19–6.30 for patients with diabetes for 10 years or more).
